# *CTNNB1-*related disorders: clinical and radiological contributions from a French cohort

**DOI:** 10.3389/fneur.2026.1754143

**Published:** 2026-02-18

**Authors:** Eline Chauvet-Piat, Marie-Céline François-Heude, Gaël Manes, Arthur Coget, Nicolas Leboucq, Bérénice Lecardonnel, Heidy Baide-Mairena, Marine Allais, Souad Touati, Stéphanie Sanchez, Mirna Khalil, Hugues Chevassus, Marjolaine Willems, David Geneviève, Marion Serrand, Laure Mazzola, Vincent Dubard, Mathilde Renaud, Caroline Le Camus, Rebecca More, Mathieu Milh, Caroline Paris, Ians-Bouteiller Cécile, Agathe Roubertie

**Affiliations:** 1 Department of Pediatric Neurology, Montpellier University Hospital, Montpellier, France; 2 Department of the Woman, Child and Adolescent, Geneva University Hospitals, Geneva, Switzerland; 3 Institute for Neurosciences of Montpellier, National Institute of Health and Medical Research (INSERM), University of Montpellier, Montpellier, France; 4 Department of Neuroradiology, Montpellier University Hospital, Montpellier, France; 5 Clinical Investigation Center Montpellier University Hospital, Montpellier, INSERM, France; 6 Department of Clinical Genetics, Arnaud de Villeneuve Hospital, Montpellier, France; 7 National Institute of Health and Medical Research (INSERM), Montpellier University Hospital, Montpellier, France; 8 Department of Pediatric Ophthalmology, Montpellier University Hospital, Montpellier, France; 9 Neurology Department, University Hospital, Saint-Etienne, France; 10 NeuroPain Lab, Lyon Neuroscience Research Centre, French National Institute of Health and Medical Research, University of Lyon, Lyon, France; 11 Department of Pediatrics, Early Medico-Social Action Center (CAMSP), Nîmes, France; 12 Clinical Genetics Department, University Hospital of Nancy, Nancy, France; 13 Neurology Department, University Hospital of Nancy, Nancy, France; 14 Faculté de Médecine, Nutrition–Génétique et Exposition aux Risques Environnementaux, Université de Lorraine, Nancy, France; 15 Department of Pediatric Neurology, Toulouse University Hospital, Toulouse, France; 16 Department of Pediatric Neurology, Charles Nicolle University Hospital, Rouen, France; 17 Pediatric Neurometabolic Department, Aix-Marseille University, Marseille, France; 18 Department of Pediatric Neurology, Jean Minjoz University Hospital, Besançon, France; 19 Department of Pediatric Neurology and Rehabilitation, Bordeaux University Hospital, Bordeaux, France

**Keywords:** cerebral palsy, CTNNB1, exudative vitreoretinopathy, intellectual disability, microcephaly, movement disorders

## Abstract

*CTNNB1* monoallelic pathogenic variants account for up to 4% of genetically determined cerebral palsy cases, yet their phenotypic spectrum remains poorly defined. We retrospectively analyzed 25 individuals with pathogenic *CTNNB1* variants using medical records and a questionnaire. Data included genetic variants, perinatal history, developmental milestones, behavioral characteristics, head growth, feeding, sleep difficulties, neurological and ophthalmological assessments. Brain MRIs were reviewed by expert neuroradiologists. Twenty-two distinct heterozygous variants were identified. Microcephaly occurred in 16/22 patients. All exhibited global developmental delay, independent walking was achieved at a mean age of 2.1 years, with regression in 4/16 independent walkers. Behavioral disorders were frequent, as were oral sensorimotor disorders (21/25) and sleep disturbances (13/21). Lower limb hypertonia was present in 22/25 patients [spastic (8) and/or dystonic (11)]. Unstable gait were common among ambulatory patients. Exaggerated startle reactions, often since birth, were reported in 16/21. Exudative vitreoretinopathy was identified in 3/5 patients with retinal angiography. Brain MRI (19 patients) showed: thickening of anterior commissure (8), frontal lobe hypoplasia (9), widening of superior vermian sulci (10) and corpus callosum anomalies (7). This study broadens the spectrum of *CTNNB1*-related syndrome, reporting a complex motor phenotype combining (i) gait disturbances related to dystonic or non-dystonic hypertonia and unsteadiness, sometimes associated to dystonia in other body parts (ii) possible deterioration of motor achievements over the course of the disease (iii) an exaggerated startle reflex. New non-specific brain anomalies are precisely described. Our work underscores the need for registries and longitudinal studies to refine characterization and guide future therapies.

## 1 Introduction

Cerebral palsy is defined as a nonprogressive neurodevelopmental disorder affecting motor development and characteristically impairs movement and posture. It is frequently accompanied by other neurodevelopmental disorders, including intellectual disability, epilepsy, and autism spectrum disorder (1).

Monoallelic pathogenic variants in the *CTNNB1* gene were first described by Ligt et al. (2) in 2012 in patients with intellectual disability associated to motor impairment. Although the true prevalence of *CTNNB1*-related syndrome remains uncertain, *CTNNB1* variants are estimated to account for up to 4% of genetically determined cases of cerebral palsy (1, 3), suggesting that many affected individuals may still go undiagnosed. The *CTNNB1* gene encodes *β*-catenin, a multifunctional protein involved in cell adhesion and transcriptional regulation within the canonical Wnt signaling pathway, which plays a critical role in cell differentiation and tissue homeostasis (4, 5). Individuals with *de novo* loss-of-function mutations in *CTNNB1* exhibit a broad phenotypic spectrum, with variable severity, which further complicates the establishment of genotype–phenotype correlations. Common clinical features include motor developmental delay, intellectual disability, microcephaly, hypotonia, motor impairments - particularly lower limb hypertonia and gait abnormalities - behavioral disturbances, distinctive ophthalmological findings, and typically no specific abnormalities on Brain Magnetic Resonance Imaging (MRI) (6–8).

We conducted a retrospective analysis of 25 French patients carrying pathogenic variants in the *CTNNB1* gene. This study provides novel insights into newly identified genetic variants, complex motor phenotype of *CTNNB1* patients, motor regression, exaggerated startle responses, and brain imaging findings, while also confirming previously described phenotypic features.

## 2 Methods

### 2.1 Clinical and radiological investigations

We conducted a retrospective study on patients with monoallelic pathogenic or likely pathogenic *CTNNB1* variants. Patients were recruited either from our existing patient cohort, through referrals from our pediatric neurology colleagues across France, or via the *CTNNB1* French association (Association *CTNNB1* France). Clinical data concerning ante-, peri- and postnatal history were collected from medical records and through a structured questionnaire, developed by the study investigators. This dedicated questionnaire was completed by families, with support from the patient’s physician, and captured the caregivers’ and referring physician’s perception of the patient’s condition. Quantitative values concerning gestational age, birth parameters, age at achievement of developmental milestones, head circumference and educational level at last follow-up, genetic variants and the diagnostic method used were collected. Other data - including pregnancy course, motor regression, learning difficulties, cognitive, behavioral and sleep disturbances, feeding difficulties and startle reactions - were collected using closed-ended categorical variables (present/absent/unknown). Results of neuropsychological assessment by standardized cognitive tools were incorporated when available.

Clinical evaluation was performed either by our team (11 patients) or by the referring physician (14 patients).

Ophthalmological abnormalities were also included among the collected data, based on both our structured questionnaire and the ophthalmology reports, covering the following variables: strabismus, refractive errors, and the presence of exudative vitreoretinopathy.

When available, brain MRIs were reviewed by 2 expert neuroradiologists (NL and AG) using a standardized and systematic reading protocol to ensure uniform assessment.

None of the patients included in this cohort had been reported in the literature prior to the present study. Data are presented as mean, standard deviation (SD) and range (minimum-maximum), when applicable, in the form mean +/− SD (range) for continuous variables, and as percentages (%) for categorical variables.

### 2.2 Identification and analysis of CTNNB1 variants

*CTNNB1* variants (RefSeq NM_001904.4) were identified using trio-based whole-exome sequencing (WES), whole-genome sequencing (WGS), targeted gene-panel sequencing or copy number variant (CNV) detection performed using chromosomal microarray and/or sequencing data. *In silico* pathogenicity prediction was performed using the MobiDetails platform,^1^ which integrates multiple algorithms. Missense variant was evaluated using ClinVar, PolyPhen-2, SIFT, MPA, ClinPred, REVEL, MISTIC, and CADD, while the splice-site variant was assessed with SPiP and SpliceAI. Population allele frequencies were verified against the Genome Aggregation Database (gnomAD, v4.1.0).

## 3 Results

**Figure 1:**
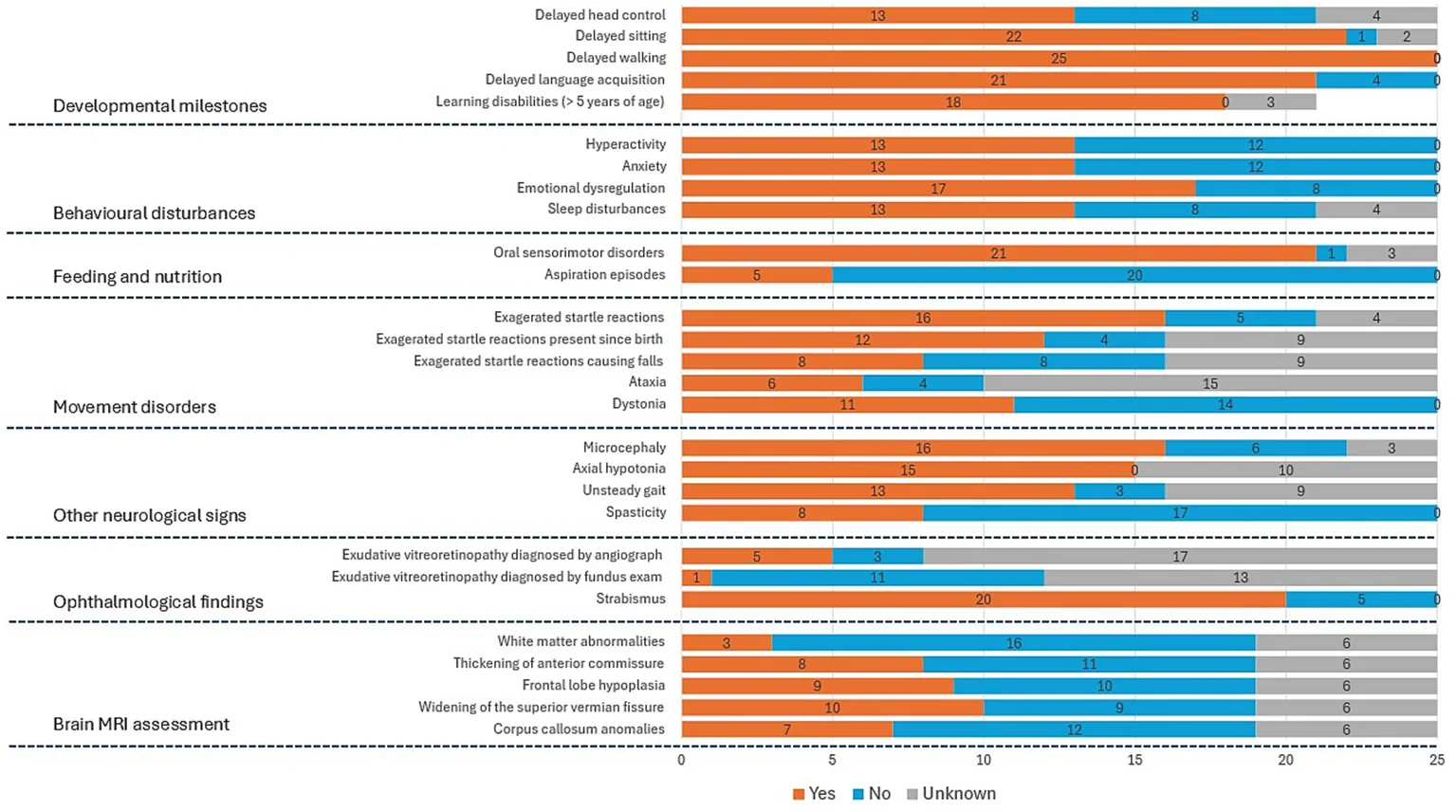
Main phenotypic features observed in our cohort of 25 patients carrying a *CTNNB1* gene variant. For each phenotypic trait, the bar chart displays the number of affected patients (in red), unaffected patients (in blue), and patients for whom the information is unavailable (in gray). The exact number of patients is indicated in black within each bar. MRI: magnetic resonance imaging.

The study included 25 unrelated patients, comprising 13 females and 12 males, mean age at last assessment: 10.9 +/− 6.7SD (range: 4.0–27 years). All but five individuals were minors (< 18 years). Unavailable data were treated as missing, and individuals were excluded from proportion calculations for that trait. The main results are summarized in Figure 1.

### 3.1 Identified genetic variants

**Figure 2:**
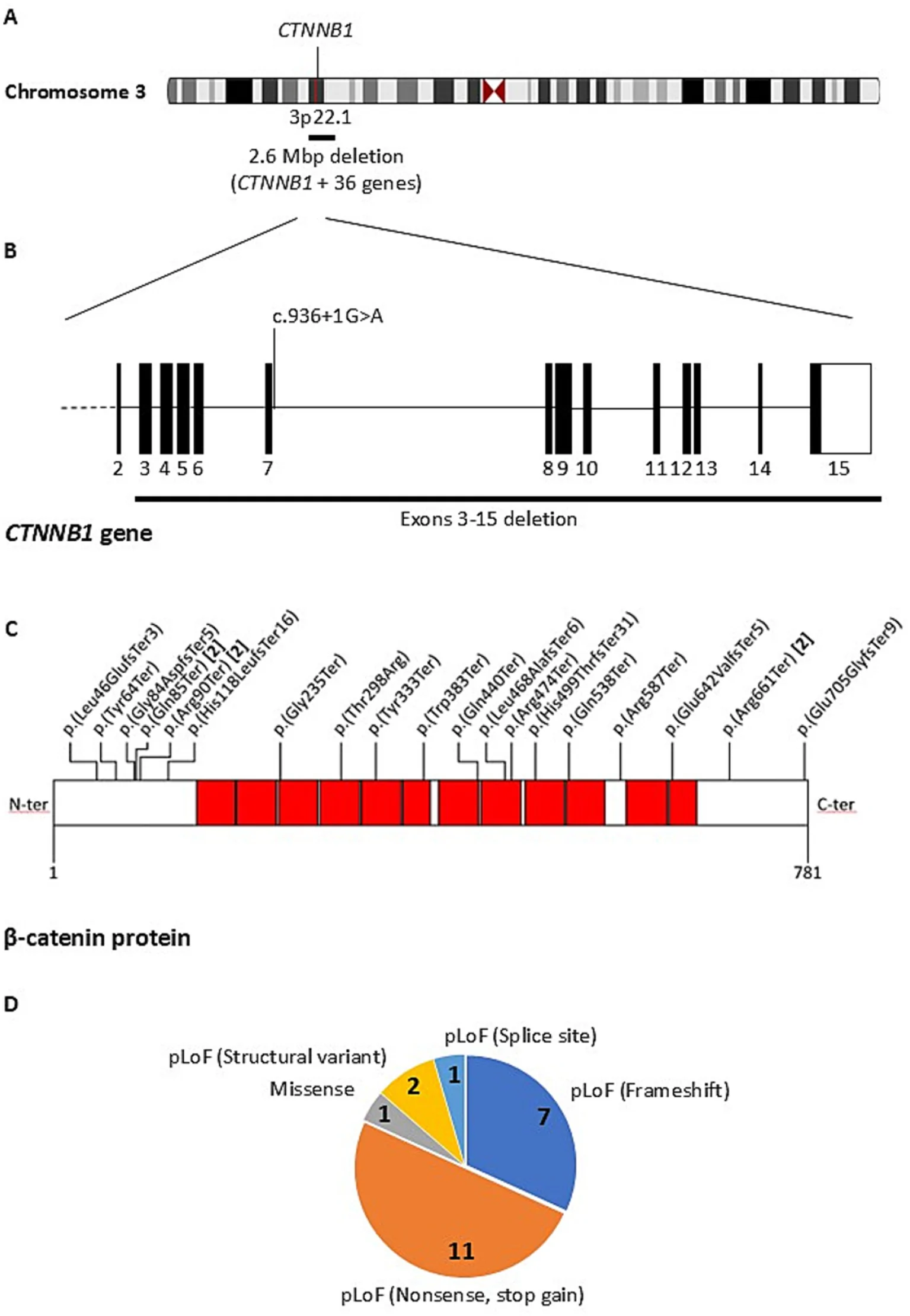
Schematic representation of the 25 *CTNNB1* pathogenic variants identified in this study. **(A)** The chromosomal localization of the *CTNNB1* gene on chromosome 3 (3p22.1) is shown, highlighting a large structural variant involving a 2.6 Mb deletion encompassing *CTNNB1* and 36 additional genes. **(B)** The exon/intron structure of the *CTNNB1* gene is represented (exon 1 not shown due to its distant position), with a deletion spanning exons 3–15 and a splice-site variant indicated. **(C)** The β-catenin protein (NP_001091679.1), encoded by *CTNNB1*, is schematically shown from N-terminus (N-ter) to C-terminus (C-ter), with its 12 armadillo repeat domains (red squares). Amino acid positions are numbered from 1 to 781. Missense, nonsense, and frameshift variants are mapped along the protein according to their location. Recurrent variants are indicated in brackets with the number of patients carrying the same change. **(D)** The distribution of the 22 distinct *CTNNB1* variants identified in this study is shown, distinguishing predicted loss-of-function (pLoF) variants (nonsense, frameshift, splice-site, and structural variants) from the single missense variant. These variants were identified in 25 unrelated patients, reflecting recurrent variants observed in different individuals. All variants are listed in Supplementary Table 1, with additional details and references.

We identified 22 distinct heterozygous *CTNNB1* variants in 25 unrelated patients, reflecting recurrent variants in different individuals (Supplementary Table 1). All but three variants were confirmed to be *de novo* by exome or genome trio sequencing, or by Sanger sequencing of the parents to assess co-segregation of the variant with the phenotype. All variants were absent from the gnomAD database. Most previously reported *CTNNB1* variants were classified as pathogenic or likely pathogenic in ClinVar, consistent with the established disease mechanism. Novel variants were interpreted as pathogenic based on ACMG criteria, predicted loss-of-function effects, and the known haploinsufficiency of *CTNNB1*. Except for a single missense variant, the remaining 21 variants were predicted to result in a loss of function (pLoF). These comprised 11 nonsense variants observed in 14 patients, seven frameshift variants leading to a premature termination codon, one splice-site variant, and two structural variants. The structural variants included a deletion of exons 3 to 15 of *CTNNB1* and a larger 2.6 Mb deletion encompassing the entire *CTNNB1* gene along with 36 other additional genes and pseudogenes (Figure 2).

The splice-site variant c.936 + 1G > A, located in intron 6, was predicted to impair the canonical donor site by SPiP (98.41% probability of loss) and SpliceAI (Donor loss score of 0.98).

The missense variant p.(Thr298Arg) was predicted to be deleterious by all *in silico* pathogenicity prediction tools used (Supplementary Table 1), but it is currently classified as a Variant of Uncertain Significance (VUS) in ClinVar, reflecting the lack of sufficient functional or clinical evidence to confirm its pathogenicity. Three nonsense variants, p.(Gln85Ter), p.(Arg90Ter), and p.(Arg661Ter), were recurrent, each identified in two unrelated individuals.

The variants were distributed across the protein without evidence of clustering within a specific hotspot region (Figure 2).

### 3.2 Pregnancy

Pregnancy was uneventful in 11 patients. Gestational diabetes was reported in 3 patients, growth retardation in 2 patients and fetal microcephaly in 1 patient (data not available in 8 patients). Prematurity was not reported. Birth weight between 2,6 to 2,7 kg was reported in 4 patients, normal birth weight in 19, while only 1 child had a birth weight under 2,6 kg (2,610 kg). Weight data was unavailable in 1.

### 3.3 Head circumference growth

Microcephaly, defined as a head circumference z-score below −2, was observed in 16 of 22 patients (72,7%). Among these, 3 cases were congenital, 10 were acquired during development. Birth head circumference data was missing for 3 patients.

### 3.4 Developmental milestones

#### 3.4.1 Head control

Delayed head control (after 4 months) was observed in 13 of 21 patients (61.9%), with a mean acquisition age of 9.3 +/−12,6 SD months.

#### 3.4.2 Sitting

Delayed sitting (after 8 months) was observed in 22 of 23 patients (95.7%), with a mean age of 20.1 +/−15.6SD months. One patient achieved sitting on time at 6 months.

#### 3.4.3 Walking

Ambulation (with or without support) was delayed (after 18 months) in every patient who achieved it: 22 of 25 patients. The remaining 3 patients (aged 3–4 years) had not yet acquired walking at last follow-up. Among the 22 walkers, 6 (27,2%) required human or device-assisted support, while 16 (72,7%) achieved independent walking, with a mean age of 2.1 +/−1.6SD years.

Among the 16 independent walkers, 4 patients (25%), harboring distinct variants, experienced regression, defined as a functional decline in walking ability, later in life: (i) A 15-year-old female patient took a few steps at age 6 but subsequently regressed with no obvious triggering factor and no longer walked at age 9 (Supplementary Table 1, patient 5), (ii) a 17-year-old patient acquired walking at 2.5 years but regressed at age 9 during a growth spurt, with increased instability and worsening right foot dystonia (Supplementary Table 1, patient 15), (iii) a 19-year-old female patient acquired walking at 3.5 years. From age 15, after right patellar dislocation, she relied exclusively on a walker (Supplementary Table 1, patient 23) (iv) a 27-year-old male patient acquired walking on tiptoes around age 4 but in his twenties, exhibited increased dependence, fear of falling, and worsening hypertonia (Supplementary Table 1, patient 25).

### 3.5 Language

Delayed language was observed in 21 of 25 patients (84%). Among these patients, the majority (12/21, 57%) exhibited a low language level for age, typically using simple sentences. Three patients (14,2%) had a very low language level, limited to isolated words with minimal word combinations, while another three (14,2%) had no functional speech, relying on pictograms or producing only syllables. Two patients achieved catch-up to age-appropriate language levels at last follow-up. Current language level is unknown for one patient.

Among the four patients without significant delay, three currently have a low language level, while only one demonstrates age-appropriate language skills.

In addition, better comprehension relative to expressive language was reported by parents in 12 patients, while comprehension data were not specified for the remaining individuals.

### 3.6 Learning and intellectual abilities

All children over 5 years of age with available data (*n* = 18) were reported to have learning disabilities (data were unavailable for 3 patients over 5 years). Among these 18 patients, eight (44,4%) attend general education settings with support or special educational program. Nine (50%) attend institutions for children with intellectual disabilities. One child is educated in a school for children with motor impairments. Intellectual disability was reported in 9 patients. Cognitive assessment through WISC-V was available for 5 patients, only one patient had a normal IQ at age 6.

### 3.7 Behavioral disturbances

Hyperactivity and anxiety were each reported in 13 of 25 patients (52%).

Emotional dysregulation was reported in 17 patients (*n* = 25, 68%), including temper outbursts and aggressive behaviors, either directed toward others, toward themselves, or both. Sleep disturbances were reported in 13 of 21 patients (61.9%), mainly involving difficulties falling asleep and/or maintaining sleep throughout the night.

### 3.8 Feeding and nutrition

Oral sensorimotor disorders were present in 21 patients (*n* = 25, 84%), including bottle feeding difficulties, food selectivity, fatigue during meals, poor weight gain, delayed solids introduction, and food rejection. Aspiration episodes occurred in 5 patients (20%). None of our patients required enteral nutritional support (through a nasogastric tube or gastrostomy).

### 3.9 Clinical evaluation

Axial hypotonia was reported in the 15 patients for whom axial tone was documented in the clinical examination.

Lower limb hypertonia was present in 22 patients (*n* = 25; 88%). When further specified, hypertonia could take the form of spasticity (8 patients) and/or dystonia (11 patients), with both patterns sometimes combined. Among the 8 with spasticity, five had brisk deep tendon reflexes and one had normal reflexes (data unavailable for two). Only two had an extensor plantar response, while three showed a flexor response (data unavailable for three). Dystonia of lower limbs was mainly distal (ankles, feet); 4 patients also had dystonia in other regions (upper limbs, neck, shoulder).

Regarding gait abnormalities, among the 22 ambulatory patients, 11 had a digitigrade gait (3 intermittently) and 3 had a plantigrade gait (gait data unavailable for 8). Among the 16 independent walkers, gait assessment was available for 13; all had unsteady gait. Ataxia was identified in 6 of 10 (60%). Notably, upper limb dysmetria was documented in only 2 of 11 patients assessed out of the total cohort of 25.

Exaggerated startle reactions were reported in 16 of 21 patients (76.2%), present since birth in 12 of 16 (75%), and potentially causing falls in 8 of 16 (50%). In depth analysis of startle reaction is currently in progress and will be reported in a future publication.

### 3.10 Ophthalmological findings

Strabismus was present in 20 of 25 patients (80%). Hypermetropia was the most common refractive impairment, affecting 17 patients (*n* = 25; 68%). Retinal evaluation was performed in 17 of 25 patients, either by fundus exam (12 patients), or fluorescein angiography (5 patients). Exudative vitreoretinopathy was diagnosed in 4 patients: 1 of 12 with fundus exam (8.3%) and 3 of 5 with angiography (60%). Two patients benefited of a retinal photocoagulation. Among the other 11 patients with fundus exam: 9 had normal findings, and 2 had optic nerve atrophy.

### 3.11 Brain MRI assessment

**Figure 3:**
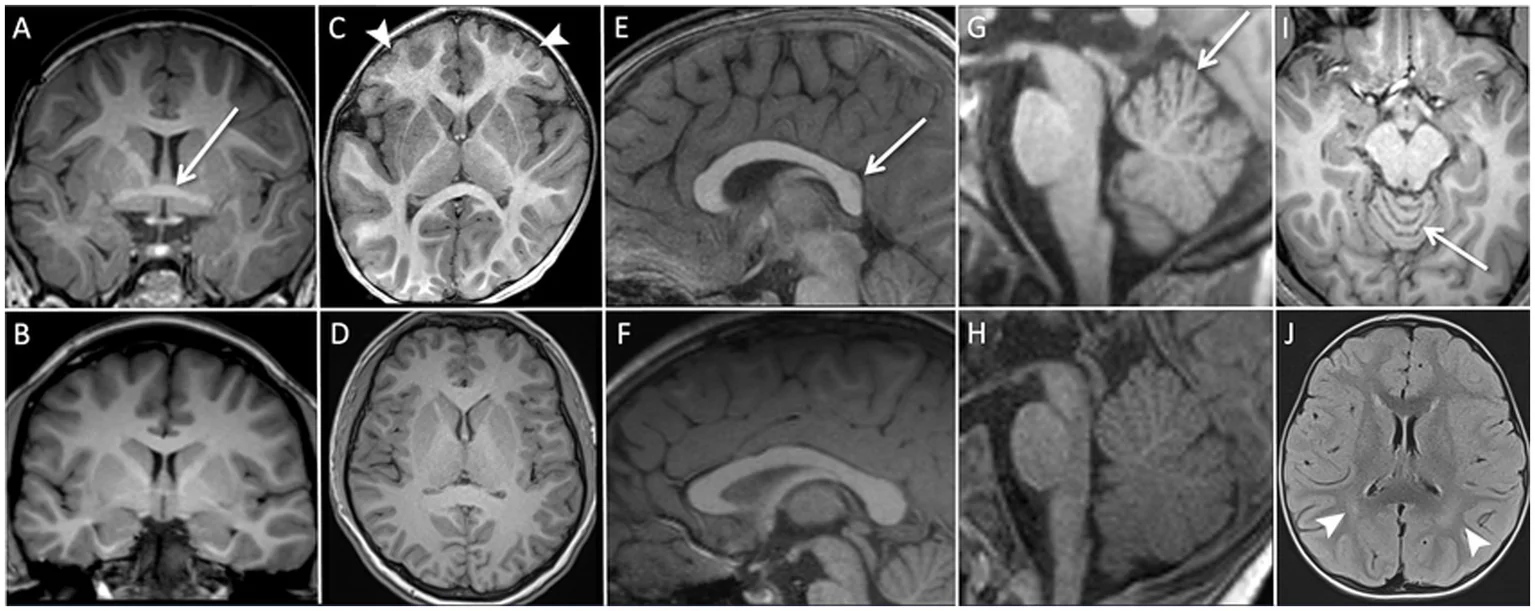
Brain MRI non-specific abnormalities. **(A)** Coronal T1-weighted sequence: thickened appearance of the anterior commissure (white arrow). **(B)** Coronal T1-weighted sequence: normal appearance of the anterior commissure. **(C)** Axial T1-weighted sequence: subtle triangular shape of the anterior cranial vault with frontal lobe hypoplasia (white arrow heads). **(D)** Axial T1-weighted sequence: normal appearance of cranial vault and frontal lobes. **(E)** Sagittal T1-weighted sequence: short corpus callosum (white arrow). **(F)** Sagittal T1-weighted sequence: normal corpus callosum. **(G)** Sagittal T1-weighted sequence: widening of the superior vermian sulci (white arrow). **(H)** Sagittal T1-weighted sequence: normal appearance of the cerebellar vermis. **(I)** Axial T1-weighted sequence: widening of the superior vermian sulci (white arrow). **(J)** Axial FLAIR sequence: periventricular leukopathy (white arrow heads).

All patients underwent brain MRI, imaging data were available for expert review in 19 cases. The mean age at which MRI was performed was 6 years (SD +/−6.3). Figure 3 illustrates the MRI findings, and comparative images of the same anatomical regions from healthy individuals have been added for improved visualization. Two MRIs (10.5%) were considered normal. Nonspecific abnormalities were frequently observed: (i) with respect to commissural abnormalities thickening of the anterior commissure was observed in 8 patients (42.1%) and corpus callosum anomalies were identified in 7 patients (36.8%), either short, thin, or both, (ii) widening of the superior vermian sulci was observed in 10 patients (52.6%). All these 10 patients were between 8 months and 17 years of age, with a mean age of 7.1 years (SD +/−6,5). (iii) Frontal lobe hypoplasia was detected in 9 patients (47.4%). Among patients with microcephaly (*n* = 12), 5 (41%) had frontal lobe hypoplasia and 7 (58%) did not. Head circumference was not available for three patients with frontal lobe hypoplasia. Three patients presented with white matter abnormalities: posterior periventricular leukopathy in two patients, and subtle FLAIR and T2 hyperintensity of the corona radiata in the third patient.

## 4 Discussion

### 4.1 Genetics

This retrospective study presents clinical and paraclinical data from 25 French patients carrying a *CTNNB1* gene variant. The cohort brings together previously unpublished cases from across France, providing a representative national sample.

In this cohort, fewer distinct *CTNNB1* variants (*n* = 22) were identified than affected patients (*n* = 25), due to recurrent variants observed in unrelated individuals. Notably, more than half of the variants were novel, further expanding the mutational spectrum of CTNNB1. The majority were predicted to result in a loss of function and were confirmed *de novo*. This mutational spectrum is consistent with previous reports establishing *CTNNB1*-related neurodevelopmental disorder as primarily caused by *de novo* truncating variants (7).

Our findings strongly support *CTNNB1* haploinsufficiency as the principal disease mechanism. The predominance of pLoF variants (nonsense, frameshift, splice-site) and large deletions is consistent with the strong loss-of-function constraint of the gene (probability of loss-of-function intolerance, *pLI* = 1.0, gnomAD v4.1.0). Only six LoF variants were observed in this database compared with 85.4 expected (observed/expected ratio = 0.07), further indicating marked intolerance to loss-of-function variation.

The single missense variant *p*. (Thr298Arg) highlights that non-truncating variants can also be pathogenic, likely through a distinct mechanism. This variant is located within the fourth armadillo repeat, a domain critical for protein–protein interactions (9). This suggests that pathogenic missense variants may disrupt interactions with binding partners, thereby altering Wnt signaling pathway activity.

Collectively, our observations highlight the need for comprehensive genetic testing of *CTNNB1* in patients with overlapping neurodevelopmental phenotypes, encompassing not only coding and splicing variants but also structural rearrangements.

### 4.2 Clinical and radiological contribution

Our findings provide new insights into the characterization of the phenotype, complementing and expanding upon previous reports in the literature. First, our study emphasizes the complex motor pattern of lower limbs dysfunction in *CTNNB1* patients. Unsteady gait was reported in all the patients with available data; lower limb hypertonia was a common feature, consistent with dystonia or described as spastic but often lacking typical pyramidal signs. Therefore, motor lower limbs’ function is characterized by a combination of unsteadiness and hypertonia, sometimes associated with dystonia involving other body parts, as suggested by Garone et al. (8). A dedicated study exploring these aspects using a gait laboratory could yield valuable insights. Second, we report functional motor deterioration, spontaneous in two patients and triggered by an intercurrent event in two patients. Garone et al. describe worsening motor signs over time (8), particularly hypertonia, and Ho et al. refer to “progressive peripheral spasticity” (10). There is little data in literature concerning regression in cerebral palsy, although age-related gait decline has been recently reported in such condition (11). A detailed longitudinal clinical observation, especially into adulthood, is needed to better characterize this new finding in *CTNNB1* patients. Third, detailed familial information collection highlighted the high prevalence of exaggerated startle reactions in more than three quarters of the patients and allowed for more precise description of their early age of onset (neonatal in 75%) and occasional impact on walking. This has been described once by Winczewska-Wiktor et al. (12) as “syndromic atypical hyperekplexia,” and by Garone et al. (8) and Nagaratnam et al. (13), following electromyogram studies, as “exaggerated startle reflex”.

Therefore, our results highlight that the *CTNNB1* spectrum is characterized by a complex motor phenotype combining (i) gait disturbances related to dystonic or non-dystonic hypertonia and unsteadiness, sometimes associated with dystonia in other body parts (ii) possible deterioration of motor achievements over the course of the disease (iii) an exaggerated startle reflex.

The additional reported phenotypic features, regarding cognition, language and motor development are in line with the current definition of cerebral palsy and are consistent with previously published descriptions. From a behavioral standpoint, both our cohort and literature converge on a high prevalence of behavioral and emotional dysregulation, including hyperactivity, anxiety, emotional instability, temper outbursts, and aggressive behaviors toward others or self (6–8, 10, 14). Autistic traits (60%) (14), attention deficits (6, 8), and even schizophrenia (4, 6, 8) have been reported occasionally. Sleep quality is generally poor, particularly regarding sleep onset and maintenance (6, 14, 15). As Sudnawa et al. (15) suggests, improving sleep quality may enhance emotional regulation, underscoring the importance of early intervention (sleep hygiene, behavioral strategies, melatonin use) (15).

With respect to radiological findings, our series contributes imaging data obtained through a systematic re-reading of the MRI examinations by expert neuroradiologists. It expands the previously described spectrum of non-specific brain abnormalities. Regarding commissural abnormalities, while hypoplasia of corpus callosum has already been described (6, 7), thickening of the anterior commissure is a novel finding. These are consistent with experimental evidence implicating *CTNNB1* (*β*-catenin) and Wnt signaling in commissural development and axonal connectivity. Both *CTNNB1* loss and gain of function perturbate midline glial cell populations in mice, disrupting the organization of guiding structures such as the glial wedge and indusium griseum glia, leading to corpus callosum dysgenesis and Probst bundle formation (16). Furthermore, Wnt signaling gradients have been shown to regulate neuronal polarization, axonal guidance and branching and synapse formation in multiple models (17). Alterations in these mechanisms likely contribute to the commissural anomalies observed in CTNNB1-related neurodevelopmental disorders, supporting a role for impaired Wnt/ *β*-catenin signaling in midline axon navigation and interhemispheric connectivity.

Hypoplasia of the frontal lobes was present in nearly half of the patients. Brain size, particularly the cerebral cortex and frontal lobes, is a major determinant of cranial size; a reduction in cortical volume is a hallmark feature of microcephaly (18, 19).

Nonspecific white matter abnormalities have already been described (6–8, 15, 20, 21). We report three patients with white matter changes. These may reflect impaired myelination due to disrupted Wnt/β-catenin signaling. This pathway is stage-dependent in oligodendrocyte development, initially inhibiting precursor formation but later required for differentiation and myelin maturation (22, 23). Future volumetric MRI studies of white matter could help detect subtle or diffuse changes not visible on conventional imaging and clarify genotype–phenotype correlations in CTNNB1-related neurodevelopmental disorders.

Exudative vitreoretinopathy is characterized by abnormal retinal vascular development. Its hallmark is a peripheral avascular retina, which may lead to complications such as neovascularization, exudation, hemorrhage, and retinal detachment (24). Early stages may be asymptomatic and detectable only through fluorescein angiography (25). In Miroševič et al. systematic review (6), most of the 22.8% of patients with exudative vitreoretinopathy had advanced disease. Sudnawa et al. (15) reported a 25% prevalence, though without specifying the diagnostic method. In our series, only 5 patients benefited of fluorescein angiography. Considering the therapeutic implication of vitreoretinopathy diagnosis, we propose that fluorescein angiography should be part of the assessment of all *CTNNB1* patients, as previously suggested by Bedoukian et al. (26).

### 4.3 Limitations

The small sample size and age heterogeneity within our cohort do not permit meaningful age-stratified comparisons - whether by age, functional level, phenotype severity, or variant type - which constitutes an inherent limitation. In this context, we consider that a pooled analysis offers the most coherent and clinically informative characterization.

Another limitation of our study is its retrospective design, with data extracted from medical records originating from multiple centers and containing numerous missing items. Moreover, the questionnaire captured caregivers’ perceptions of the patient’s condition, which introduces intrinsic inter-individual assessment bias. Nevertheless, this approach also highlights the daily -life burden of the disease as experienced by families and reported by referring physicians. Establishing a patient registry and conducting a prospective longitudinal assessment would be crucial to better characterize developmental and head growth trajectories, potential regression patterns and detailed natural history of *CTNNB1* disease. Moreover, standardized clinical scale for *CTNNB1*-related disorders must be elaborated as it will be essential for development of innovative therapies, including targeted pharmacological treatments and gene therapy.

## Data Availability

The datasets presented in this study can be found in online repositories. The names of the repository/repositories and accession number(s) can be found in the article/Supplementary material.

## Footnotes

1
https://mobidetails.chu-montpellier.fr/
