# Supplementary material for: CTNNB1-related disorders: clinical and radiological contributions from a French cohort
Source: Front Neurol. 2026 Feb 18;17:1754143. doi: 10.3389/fneur.2026.1754143 (PMC12956669; doi:10.3389/fneur.2026.1754143)
Supplement: Supplementary file 1 [file Table_1.docx]

CTNNB1-Related Disorders: Clinical and Radiological Contributions from a French Cohort

Supplementary Table 1. Summary of CTNNB1 (catenin beta 1, transcript NM_001904.4) heterozygous variants reported in the present study

| Patient | Nucleotide  change | Protein  change | Exon | Variant type | Inheritance | gnomAD | MPA score | ClinVar | SIFT | PPhen2 | Mistic | ClinPred | REVEL | CADD | SPiP  SpliceAI | Reference | Comments |
| --- | --- | --- | --- | --- | --- | --- | --- | --- | --- | --- | --- | --- | --- | --- | --- | --- | --- |
| P1 | c.136_137del | p.(Leu46GlufsTer3) | 3 | pLoF – frameshift | De novo | 0 | 10 | Nomatch | NA | NA | NA | NA | NA | NA | NA | Not reported |  |
| P2 | c.192T>A | p.(Tyr64Ter) | 3 | pLoF – nonsense | De novo | 0 | 10 | No match | NA | NA | NA | NA | NA | NA | NA | Not reported |  |
| P3 | c.251del | p.(Gly84AspfsTer5) | 4 | pLoF – frameshift | De novo | 0 | 10 | No match | NA | NA | NA | NA | NA | NA | NA | Not reported |  |
| P4 | c.253C>T | p.(Gln85Ter) | 4 | pLoF – nonsense | De novo | 0 | 10 | PAT | NA | NA | NA | NA | NA | NA | NA | Not reported | Recurrent (n=2) in cohort |
| P5 | c.253C>T | p.(Gln85Ter) | 4 | pLoF – nonsense | De novo | 0 | 10 | PAT | NA | NA | NA | NA | NA | NA | NA | Not reported | Recurrent (n=2) in cohort |
| P6 | c.268C>T | p.(Arg90Ter) | 4 | pLoF – nonsense | De novo | 0 | 10 | PAT | NA | NA | NA | NA | NA | NA | NA | (1) | Recurrent (n=2) in cohort |
| P7 | c.268C>T | p.(Arg90Ter) | 4 | pLoF – nonsense | De novo | 0 | 10 | PAT | NA | NA | NA | NA | NA | NA | NA | (1) | Recurrent (n=2) in cohort |
| P8 | c.351_352dup | p.(His118LeufsTer16) | 4 | pLoF – frameshift | Unconfirmed | 0 | 10 | No match | NA | NA | NA | NA | NA | NA | NA | Not reported |  |
| P9 | c.703G>T | p.(Gly235Ter) | 5 | pLoF – nonsense | De novo | 0 | 10 | No match | NA | NA | NA | NA | NA | NA | NA | Not reported |  |
| P10 | c.893C>G | p.(Thr298Arg) | 6 | Missense | De novo | 0 | 9.0 | VUS | 0.001 DAM | 1.0 PRO | 0.93 DAM | 0.994 DAM | 0.797 DAM | 29.80 | NA | (2) | Reported in a patient with ASD |
| P11 | c.936+1G>A | p.(?) | Intr. 6 | pLoF – splice | De novo | 0 | 10 | PAT | NA | NA | NA | NA | NA | NA | 98.41% 0.98 | Not reported |  |
| P12 | c.998dup | p.(Tyr333Ter) | 7 | pLoF – nonsense | De novo | 0 | 10 | PAT | NA | NA | NA | NA | NA | NA | NA | (3) |  |
| P13 | c.1148G>A | p.(Trp383Ter) | 8 | pLoF – nonsense | De novo | 0 | 10 | PAT | NA | NA | NA | NA | NA | NA | NA | (4) |  |
| P14 | c.1318C>T | p.(Gln440Ter) | 9 | pLoF – nonsense | De novo | 0 | 10 | LIK | NA | NA | NA | NA | NA | NA | NA | Not reported |  |
| P15 | c.1316_1401dup | p.(Leu468AlafsTer6) | 9 | pLoF – frameshift | De novo | 0 | NA | NA | NA | NA | NA | NA | NA | NA | NA | Not reported |  |
| P16 | c.1420C>T | p.(Arg474Ter) | 9 | pLoF – nonsense | De novo | 0 | 10 | PAT | NA | NA | NA | NA | NA | NA | NA | (4) |  |
| P17 | c.1494dup | p.(His499ThrfsTer31) | 9 | pLoF – frameshift | De novo | 0 | 10 | PAT | NA | NA | NA | NA | NA | NA | NA | (5) |  |
| P18 | c.1612C>T | p.(Gln538Ter) | 10 | pLoF – nonsense | De novo | 0 | 10 | PAT | NA | NA | NA | NA | NA | NA | NA | (6) |  |
| P19 | c.1759C>T | p.(Arg587Ter) | 11 | pLoF – nonsense | De novo | 0 | 10 | PAT | NA | NA | NA | NA | NA | NA | NA | (7) |  |
| P20 | c.1925_1926del | p.(Glu642ValfsTer5) | 12 | pLoF – frameshift | De novo | 0 | 10 | PAT | NA | NA | NA | NA | NA | NA | NA | Not reported |  |
| P21 | c.1981C>T | p.(Arg661Ter) | 13 | pLoF – nonsense | Unconfirmed | 0 | 10 | PAT | NA | NA | NA | NA | NA | NA | NA | (6) | Recurrent (n=2) in cohort |
| P22 | c.1981C>T | p.(Arg661Ter) | 13 | pLoF – nonsense | Unconfirmed | 0 | 10 | PAT | NA | NA | NA | NA | NA | NA | NA | (6) | Recurrent (n=2) in cohort |
| P23 | c.2113dup | p.(Glu705GlyfsTer9) | 14 | pLoF – frameshift | De novo | 0 | 10 | Nomatch | NA | NA | NA | NA | NA | NA | NA | Not reported |  |
| P24 | Exons 3-15 deletion | null allele | 3-15 | pLoF – SV | De novo | 0 | NA | NA | NA | NA | NA | NA | NA | NA | NA | Not reported |  |
| P25 | 2.6 Mb deletion | null allele | 1-15 | pLoF – SV | De novo | 0 | NA | NA | NA | NA | NA | NA | NA | NA | NA | Not reported |  |

**Footnotes:**
gnomAD; (Genome Aggregation Database)*,* Allele frequency in the gnomAD v4.1.0 database.

pLoF, predicted to result in a loss of function (pLoF)

pLoF (frameshift with premature stop codon), pLoF – frameshift

pLoF (nonsense, stop gain), pLoF – nonsense

pLoF (splice site), pLoF – splice

pLoF (structural variant), pLoF – SV

MPA; (MoBiDiC Prioritization Algorithm), scores range from 1 to 10, the higher the score, the more likely the variant has damaging effect

ClinVar; Public archive of variant–phenotype relationships, classified as Pathogenic (PAT), Likely Pathogenic (LIK), Uncertain (VUS), Likely Benign (LBEN), or Benign (BEN); no match: variant not reported in ClinVar as of october 2025.

SIFT; (Sorting Intolerant from Tolerant), scores range from 0.0 (Damaging, DAM) to 1.0 (Tolerated, TOL)

PPhen2; polymorphism Phenotyping v2 (HumDiv model), scores range from 0.0–0.15 (Benign, BEN), 0.15–0.85 (Possibly damaging, POS), 0.85–1.0 (Probably damaging, PRO)

Mistic; (MISsense deleTeriousness predICtor), scores range from 0 to 1; ≥0.5 for damaging (DAM)

ClinPred; Prediction tool for disease-relevant nonsynonymous single nucleotide variants, ≥ 0.5 for damaging (DAM), <0,5 for benign (BEN)

REVEL; (Rare Exome Variant Ensemble Learner), scores range from 0 to 1, DAM for damaging, UNC for uncertain, BEN for benign.

CADD; (Combined Annotation Dependent Depletion), >30 highly pathogenic, >20 pathogenic (PAT), 15-20 likely pathogenic (LIK), <15 likely benign (BEN)

SPiP, (Splicing Prediction Pipeline), probability of splicing alteration; ≥0.8 indicates high confidenc,

SpliceAI Donor Loss; Deep-learning–based score predicting donor splice site disruption; 0 (no effect) to 1 (complete loss); ≥0.8 highly deleterious
ASD: Autism Spectrum Disorder

NA: Not Applicable

Int. for intron

References:

1. Žakelj N, Gosar D, Miroševič Š, Sanders SJ, Ljungdahl A, Kohani S, et al. Genotypic, functional, and phenotypic characterization in CTNNB1 neurodevelopmental syndrome. Hum Genet Genomics Adv. 18 juill 2025;6(4):100483.

2. Al-Mamari W, Idris AB, Al-Thihli K, Abdulrahim R, Jalees S, Al-Jabri M, et al. Applying whole exome sequencing in a consanguineous population with autism spectrum disorder. Int J Dev Disabil. 69(2):190‑200.

3. Sinibaldi L, Garone G, Mandarino A, Iarossi G, Chioma L, Dentici ML, et al. Congenital heart defects in CTNNB1 syndrome: Raising clinical awareness. Clin Genet. nov 2023;104(5):528‑41.

4. Lee S, Jang SS, Park S, Yoon JG, Kim SY, Lim BC, et al. The extended clinical and genetic spectrum of CTNNB1-related neurodevelopmental disorder. Front Pediatr. 2022;10:960450.

5. Yan D, Sun Y, Xu N, Yu Y, Zhan Y. Genetic and clinical characteristics of 24 mainland Chinese patients with CTNNB1 loss‐of‐function variants. Mol Genet Genomic Med. 24 sept 2022;10(11):e2067.

6. Miroševič Š, Khandelwal S, Sušjan P, Žakelj N, Gosar D, Forstnerič V, et al. Correlation between Phenotype and Genotype in CTNNB1 Syndrome: A Systematic Review of the Literature. Int J Mol Sci. 19 oct 2022;23(20):12564.

7. Yoo Y, Jung J, Lee YN, Lee Y, Cho H, Na E, et al. GABBR2 mutations determine phenotype in rett syndrome and epileptic encephalopathy. Ann Neurol. sept 2017;82(3):466‑78.
